# Mitochondrial modulators in the treatment of bipolar depression: a systematic review and meta-analysis

**DOI:** 10.1038/s41398-021-01727-7

**Published:** 2022-01-10

**Authors:** Liang Liang, Junyu Chen, Ling Xiao, Qing Wang, Gaohua Wang

**Affiliations:** 1 Department of Psychology, The Fourth Affiliated Hospital of Xinjiang Medical University, Urumqi, Xinjiang China; 2 Department of Psychiatry, Renmin Hospital of Wuhan University, Hubei Zhang Road (formerly Ziyang Road), Wuchang District No. 99, Jiefang Road 238, Wuhan, Hubei China

**Keywords:** Bipolar disorder, Clinical pharmacology

## Abstract

Mitochondrial dysfunction has been implicated in the risk, pathophysiology, and progression of mood disorders, especially bipolar disorder (BD). Thus, the objective of this meta-analysis was to determine the overall antidepressant effect of mitochondrial modulators in the treatment of bipolar depression. Outcomes included improvement in depression scale scores, Young Mania Rating Scale (YMRS) and Clinical Global Impression-Severity Scale (CGI-S) score. Data from randomized controlled trials (RCTs) assessing the antidepressant effect of diverse mitochondrial modulators were pooled to determine standard mean differences (SMDs) compared with placebo.13 RCTs were identified for qualitative review. The overall effect size of mitochondrial modulators on depressive symptoms was −0.48 (95% CI: −0.83 to −0.14, *p* = 0.007, *I*^2^ = 75%), indicative of a statistically significant moderate antidepressant effect. In the subgroup analysis, NAC improved depressive symptoms compared with placebo (−0.88, 95% CI: −1.48 to −0.27, *I*^2^ = 81%). In addition, there was no statistical difference between mitochondrial modulators and placebo in YMRS. Although mitochondrial modulators were superior to placebo in CGI-S score (−0.44, 95% CI: −0.83 to −0.06, *I*^2^ = 71%), only EPA was superior to placebo in subgroup analysis. Overall, a moderate antidepressant effect was observed for mitochondrial modulators compared with placebo in the treatment of bipolar depression. The small number of studies, diversity of agents, and small sample sizes limited interpretation of the current analysis.

## Introduction

Bipolar disorder (BD) is a common mood disorder characterized by alternating manic and depressive episodes [1]. Previous study reported that patients with bipolar depression are four times more likely to commit suicide compared to the general population [2]. Current psychopharmacological therapies are often insufficient, and about 40% of people with BD do not adhere to their prescribed treatment [3]. Therefore, there is an urgent need to elucidate novel targets that may yield improved efficacy and prevent subsequent mood episodes.

Although antidepressants may be used in combination with mood stabilizers or second-generation antipsychotics for bipolar depression, the treatment of BD is still particularly challenging because of the high non-responder rate. One treatment avenue currently being explored is the adjunctive use of mitochondrial modulators [4]. Several studies showed that mitochondrial dysfunction and oxidative stress may be involved in the development and progression of BD [5–7]. Mitochondria regulate energy production and generation of adenosine-5′-triphosphate (ATP) through the mitochondrial electron transport chain (ETC). Furthermore, they also regulate calcium and apoptotic processes and are central to facilitating neuronal plasticity. Therefore, dysfunctional mitochondria can result in neuronal damage via multiple mechanisms. At present, there are N-acetyl-cysteine (NAC), acetyl-l-carnitine (ALCAR), S-adenosylmethionine (SAMe), coenzyme Q10 (CoQ10), alpha-lipoic acid (ALA), creatine monohydrate (CM), vitamin D, and melatonin exist for treating bipolar depression [4, 8]. In the last few decades, substantial efforts have been made to evaluate the efficacy of mitochondrial modulators for the treatment of bipolar depression, both in monotherapy and as adjunctive treatment [9–21]. However, it remains unclear whether mitochondrial modulators has any benefit for the treatment of depressive symptoms in patients with mood disorders. For example, Kishi’s meta-analysis reported that N-acetylcysteine decreased CGI-S score, but no specific improvements in depressive symptoms [22]. However, Rosenblat’s meta-analysis showed that a moderate antidepressant effect was observed for adjunctive N-acetylcysteine in patients with mood disorders [23]. In addition, other mitochondrial regulators are also controversial in the treatment of depressive symptoms.

This meta-analysis was conducted to examine efficacy of mitochondrial modulators for the treatment of BDs in patients with depressive symptoms.

## Methods

This meta-analysis was performed according to the Preferred Reporting Items for Systematic Reviews and Meta-Analysis (PRISMA) guidelines [24].

### Search methods for identification of trials

Two authors independently identified eligible studies indexed in the PubMed, MEDLINE, Cochrane, and Embase databases published in any language from the inception of the study to October 25, 2020. The search terms included (bipolar depression or BD) and (N-acetylcysteine or omega-3 polyunsaturated fatty acids or inositol or CoQ10 or ALA or CM or vitamin D). The authors also searched ClinicalTrials.gov (http://clinicaltrials.gov/) to ensure a comprehensive search. The reference lists of the retrieved publications were searched manually for additional relevant studies. All identified articles were screened by two independent reviewers for inclusion in qualitative and quantitative analysis. Where there was disagreement on inclusion, the consensus was reached through discussion.

### Inclusion criteria

Inclusion criteria are as follows: (1) participants over the age of 18 years; (2) DSM or ICD diagnosis of bipolar I disorder (BD-I), bipolar II (BD-II) disorder or BD not otherwise specified (BD-NOS); (3) RCTs of mitochondrial modulators compared with placebo; (4) depression severity assessed and reported using Montgomery and Asberg Depression Rating Scale (MADRS) or Hamilton Depression Rating Scale (HDRS). The authors were contacted for data not provided in the papers. If the authors could not provide the necessary data, the trial was excluded from the quantitative analysis.

### Data extraction and statistical analysis

Two authors performed independently to identify RCTs that met the inclusion criteria and then data (including study characteristics, risks of bias, and depression severity scores) were extracted from included studies. Changes in depression severity scores (MADRS and HDRS), Young Mania Rating Scale (YMRS) scores, and Clinical Global Impression-Severity Scale (CGI-S) scores of mitochondrial modulators treatment versus placebo were used in the analysis. A prespecified *p*-value of 0.05 was set to determine the presence of a statistically significant reduction in depression severity. To further evaluate the eligibility of potential studies, we discussed any disagreements with another author.

The meta-analysis was performed using the RevMan version 5.3. Continuous outcomes were analyzed by calculating the SMDs with 95% CIs. Pooled effect sizes were subgrouped based on the mitochondrial modulator tested and then pooled to calculate the overall effect size of all mitochondrial modulators included. The statistical heterogeneity and inconsistency in treatment effects across studies were evaluated using Cochran’s *Q* test and *I*^2^ statistics, respectively. Heterogeneity was quantified using the *I*^2^ statistic, where 25% = small, 50% = moderate, and 75% = high heterogeneity [25]. Statistical significance was defined as *p* < 0.05.

### Assessment of bias

The risk of bias was assessed using the tool recommended by the Cochrane Handbook for systematic reviews of randomized trials. The risks of selection bias, performance bias, detection bias, attrition bias, reporting bias and other biases were independently examined by two reviewers and categorized as low risk, high risk, or unclear risk. The risk of bias was designated to be high if described protocols were concerning for bias in a given domain or if the description of the domain was omitted from the primary text. In addition, to assess for publication bias, a funnel plot was created using Review Manager 5.3 Software.

## Results

### Search results

**Fig. 1:**
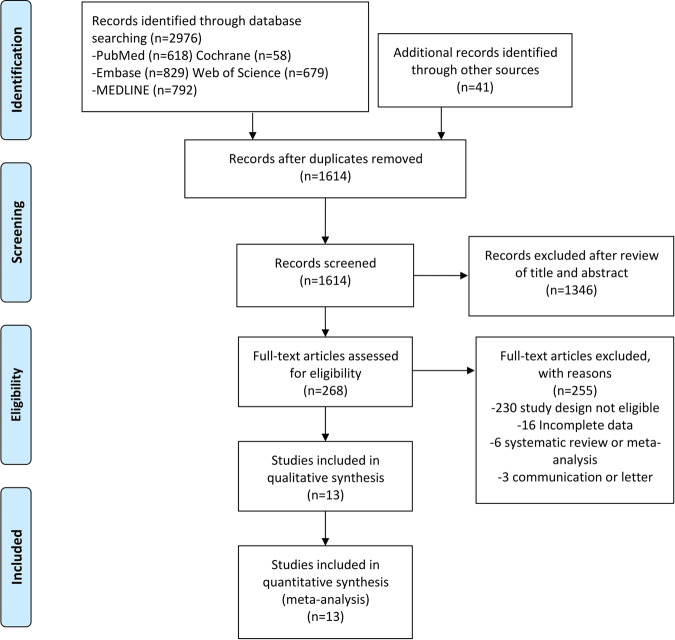
PRISMA flow diagram. Preferred Reporting Items for Systematic Reviews and Meta-Analyses (PRISMA) study selection flow diagram.

A total of 3017 records were identified, 2976 through database searches, and 41 through other sources. Of these articles, 1403 were deemed ineligible after thoroughly screening their titles and abstracts. The remaining 268 studies underwent a full-text evaluation to further evaluate their eligibility in which 255 articles were excluded because they did not meet the inclusion criteria. Ultimately, a total of 13 studies were included in this meta-analysis, including four RCTs of N-acetylcysteine (NAC) [9–12], three RCTs of Omega-3 polyunsaturated fatty acids (EPA) [13–15], one RCT of Coenzyme Q10 (CoQ10) [16], one RCT of CM [17], one RCT of Vitamin D [20], two RCTs of Inositol [18, 19], and one RCT of ALCAR+ALA [21] (Fig. 1). Table 1 shows the characteristics of each study and the characteristics of patients included in each study.

**Table 1:** Summary of study characteristics, demographics and treatment characteristics of the double-blinded, randomized, placebo-controlled trials.

Study	Studylength, weeks	Diagnostic criteria	Adjunctivea agent and dosage (*n*)	Gender, female, *n* (%)	Age, years, mean ± SD	Outcome measures
Berk et al. [9]	24w	BDI or BDII (DSM-IV)	NAC 2 g/day (38) PLA (37)	60	45.6 ± 12.5	MADRS, YMRS, CGI-S
Magalhães et al. [10]	24w	BDI or BDII (DSM-IV)	NAC 2 g/day (10) PLA (7)	53.5	42.9 ± 15.4	MADRS, YMRS
Berk et al. [11]	16w	BDI, BDII or BD-NOS (DSM-IV-TR), MADRS ≧ 20	NAC 2 g/day (59) PLA (61)	70	45.2 ± 12.1	MADRS, HDRS, YMRS, CGI-S
Ellegaard et al. [12]	20w	BDI or BDII (DSM-IV), MADRS ≧ 20	NAC 3 g/day (40) PLA (40)	59	43.4 ± 10.1	MADRS, YMRS, CGI-S
Frangou et al. [14]	12w	BDI or BDII (DSM-IV), HDRS-17 > 10	EPA 2 g/day (25) PLA (26)	70.3	47.9 ± 10.8	HDRS, YMRS, CGI-S
Stoll et al. [13]	16w	BDI (DSM-IV)	EPA 6.2 g/day + DHA 3.2 g/day (14) PLA (16)	66.5	43.0 ± 8.6	HDRS, YMRS, CGI-S
Hirashima et al. [15]	4w	BDI (DSM-IV)	EPA 1.3–5.2 g/day + DHA 0.7–3.4 g/day (12) PLA (9)	100	33.1 ± 10.0	HDRS, YMRS
Mehrpooya et al. [16]	8w	BDI, BDII or BD-NOS DSM-V),MADRS ≧ 15	CoQ10 200 mg/day (36) PLA (33)	84.1	38.5 ± 10.8	MADRS
Toniolo et al. [17]	6w	BDI or BDII (DSM-IV), MADRS ≧ 20	CM 6 g/day (16) PLA (11)	73.9	43.8 ± 9.3	MADRS, HDRS, YMRS, CGI-S
Marsh et al. [20]	12w	BDI, BDII or BD-NOS (DSM-IV)	Vitamin D 5000 IU/day (16) PLA (17)	48.1	44.2 ± 13.1	MADRS, HDRS
Chengappa et al. [18]	6w	BDI or BDII (DSM-IV), HDRS ≧ 15	Inositol 12 g/day (12) PLA (12)	63.6	42.5 ± 10.5	MADRS, HDRS, CGI-S
Evins et al. [19]	6w	BDI or BDII (DSM-IV), HDRS ≧ 15	Inositol 13.87 g/day (9) PLA (8)	64.7	45.7 ± 12.2	HDRS, CGI-S, YMRS
Brennan et al. [21]	12w	BDI or BDII (DSM-IV), MADRS ≧ 20	Acetyl-L carnitine 1000–3000 mg + a-lipoic acid 600–1800 mg/day (20) PLA (20)	67.5	45.5 ± 11.1	MADRS, HDRS, YMRS, CGI-S

*BD-I* bipolar I disorder, *BD-II* bipolar II disorder, *BD-NOS* bipolar disorder not otherwise specified, *CGI-S* clinical global impression-severity, *CoQ10* coenzyme Q10, *CM* creatine monohydrate, *DSM* diagnostic and statistical manual of mental disorders, *EPA* eicosapentaenoic acid, *HDRS* Hamilton Depression Rating Scale, MADRS Montgomery–Asberg Depression Rating Scale, *NAC* N-acetylcysteine, *YMRS* Young Mania Rating Scale.

### Assessment of bias

**Fig. 2:**
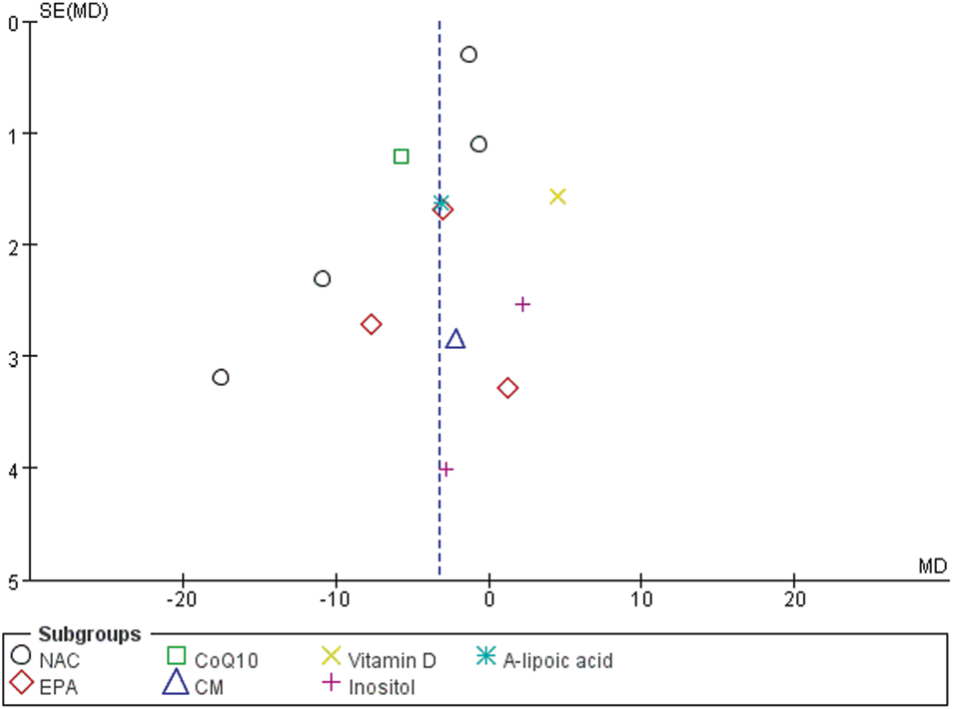
Funnel plot for publication bias. Begg’s funnel plot for publication bias analysis.

Included studies were assessed for bias in seven domains—namely, random sequence generation, allocation concealment, blinding of participants and personnel, blinding of outcome assessors, incomplete outcome data, selective reporting, and other biases. The results are summarized in Table 2. All included studies were assessed independently by two authors, and the risk of bias for each item was categorized as ‘low risk’, ‘unclear’ or ‘high risk’. Publication bias was assessed using a funnel plot, as shown in Fig. 2.

**Table 2:** Assessment of risk of bias.

Study	Sequence generation	Allocation concealment	Blinding of participants and personnel	Blinding of outcome assessors	Incomplete outcome data	Selective reporting	Other bias
Berk et al. [9]	Low	Low	Low	Low	Low	Low	Low
Magalhães et al. [10]	Low	Low	Low	Low	Low	Low	Low
Berk et al. [11]	Low	Low	Low	Low	Low	Low	Low
Ellegaard et al. [12]	Low	Low	Low	Low	Low	Low	Low
Frangou et al. [14]	Low	Low	Low	Low	Low	Low	High
Stoll et al. [13]	Low	Low	Low	Low	Low	Low	Low
Hirashima et al. [15]	High	High	High	High	Low	Low	Low
Mehrpooya et al. [16]	Low	Low	Low	Low	Low	Low	Low
Toniolo et al.(2018)	Low	Low	Low	Low	Low	Low	Low
Marsh et al. [20]	Low	Low	Low	Low	Low	Low	Low
Chengappa et al. [18]	Low	Low	Low	Low	Low	Low	Low
Evins et al. [19]	Low	Low	Low	Low	Low	Low	Low
Brennan et al. [21]	Low	Low	Low	Low	Low	Low	Low

### Change in depression severity scores

**Fig. 3:**
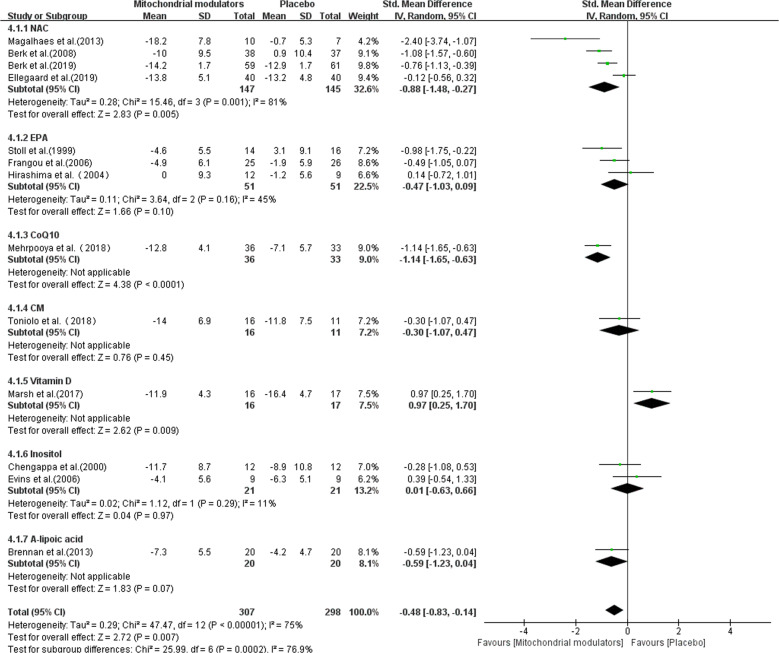
Forest plot of pooled effect sizes of mitochondrial modulators for bipolar depression. CoQ10 Coenzyme Q10, CM creatine monohydrate, EPA eicosapentaenoic acid, NAC N-acetylcysteine, SD standard deviation, SMD standard mean difference.

The pooled effect size was based on a total of 605 participants, including studies assessing NAC (*n* = 292), EPA (*n* = 102), CoQ10 (*n* = 69), CM (*n* = 27), vitamin D (*n* = 33), Inositol (*n* = 42), ALCAR+ALA (*n* = 40). As shown in Fig. 3, the overall SMD of mitochondrial modulators compared with placebo was −0.48 (95% CI: −0.83 to −0.14, *p* = 0.007, *I*^2^ = 75%), indicative of a statistically significant moderate antidepressant effect. In the subgroup analysis, four RCTs assessed the antidepressant effects of NAC in patients with BD. The results showed an SMD of −0.88 (95% CI: −1.48 to −0.27, *I*^2^ = 81%). Three studies using EPA in the treatment of BD were identified. Pooling of effect sizes for these studies revealed an SMD of −0.47 (95% CI: −1.03 to 0.09, *I*^2^ = 45%), indicative of no statistical difference between EPA and placebo. Two studies using inositol in the treatment of BD were identified. The calculated effect size was 0.01 (95% CI: −0.63 to 0.66, *I*^2^ = 11%), indicative of no statistical difference between inositol and placebo. One RCT investigating the effect of CoQ10 was identified. A significantly greater reduction in MADRS scores was observed in the CoQ10 group compared with the placebo group. However, there were no statistically significant differences observed in the CM and ALCAR+ALA groups compared with the placebo. In addition, one notable trial assessed the antidepressant effect of vitamin D was negative for this study.

### Change in YMRS

**Fig. 4:**
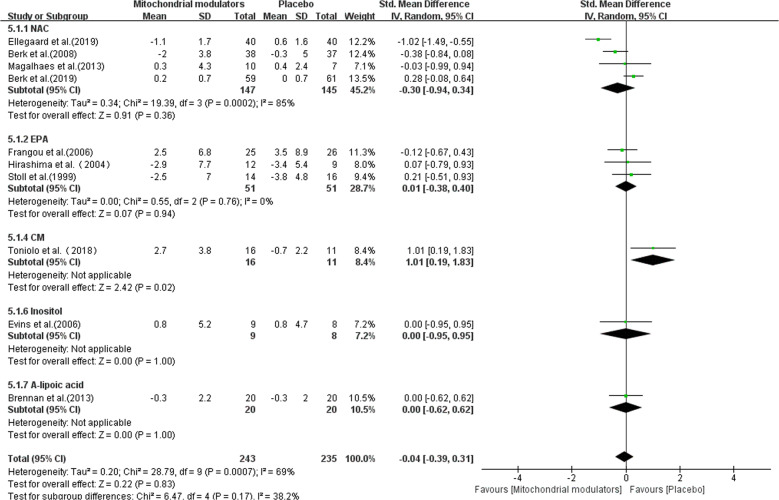
Forest plots for YMRS reductions with mitochondrial modulators. CM creatine monohydrate, EPA eicosapentaenoic acid, NAC N-acetylcysteine, SD standard deviation, SMD standard mean difference.

The pooled effect size of YMRS was based on a total of 478 including studies assessing NAC (*n* = 292), EPA (*n* = 102), CM (*n* = 27), Inositol (*n* = 17), ALCAR + ALA (*n* = 40). As shown in Fig. 4, the overall SMD of mitochondrial modulators compared with placebo was −0.04 (95% CI: −0.39 to 0.31, *I*^2^ = 69%), indicative of no statistical difference between mitochondrial modulators and placebo. In addition, there were no differences in the results of each subgroup analysis.

### Change in CGI-S

**Fig. 5:**
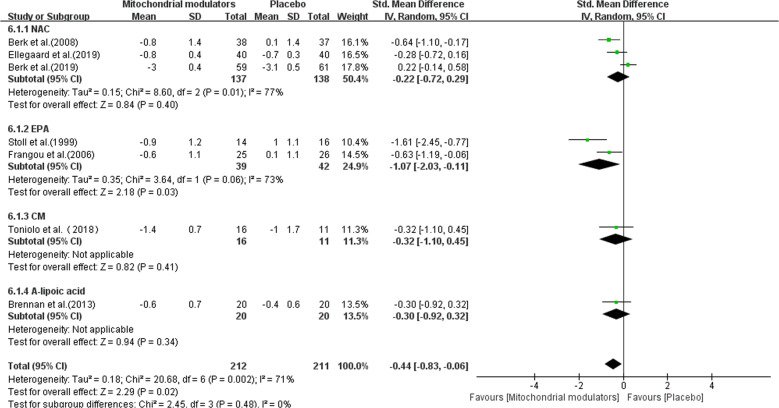
Forest plots for CGI-S reductions with mitochondrial modulators. CM creatine monohydrate, EPA eicosapentaenoic acid, NAC N-acetylcysteine, SD standard deviation, SMD standard mean difference.

The pooled effect size of CGI-S was based on a total of 423 including studies assessing NAC (*n* = 275), EPA (*n* = 81), CM (*n* = 27), ALCAR + ALA (*n* = 40). For the CGI-S, the overall SMD of mitochondrial modulators compared with placebo was −0.44 (95% CI: −0.83 to −0.06, *I*^2^ = 71%), indicative of a statistically significant difference. In the subgroup analysis, only EPA was superior to placebo in CGI-S (−1.07, 95% CI: −2.03 to −0.11, *I*^2^ = 73%) (Fig. 5).

## Discussion

To our knowledge, this is the first systematic review to evaluate the efficacy of mitochondrial modulators in the treatment of bipolar depression. This analysis suggests that mitochondrial modulators have a significant antidepressant effect in BD when compared with placebo (as measured by the change in depressive symptom severity). In this study, the overall effect size was found to be moderate (SMD = −0.48), and comparable with the antidepressant effect size of quetiapine (SMD = −0.29), lurasidone (SMD = −0.36), olanzapine (SMD = −0.52), as indicated by a previous meta-analysis [26]. Our results also showed that no significant change between each drug in YMRS. In addition, the results highlighted that mitochondrial modulators decreased CGI-S scores (assessing overall disease severity in patients) compared with the placebo.

Subgroup analysis of EPA, CM, inositol, ALCAR+ALA, and vitamin D underpowered, revealed effect sizes that were not statistically significant. Only NAC and CoQ10 were found to have a statistically significant antidepressant effect; however, the effect analysis of CoQ10 was based only on a single study, rather than a pooled sample. Our finding was consistent with a meta-analysis of NAC in depression by Fernandes et al. [27]. However, another study did not support the use of N-acetylcysteine as an adjunct to usual treatment for patients with depressive symptoms [22]. In summary, this study suggests that mitochondrial modulators may have antidepressant properties in BD, although statistical significance was only reached when pooling the effects of all mechanistically dissimilar agents together.

This study has certain limitations. First, there was a limited number of studies and small sample sizes in this meta-analysis. No associations with respect to the primary outcome and other factors were found. All sensitivity analyses also showed no reduction in *I*^2^ values below 50%. Thus, we did not detect any reasons for considerable heterogeneity in the results of the primary outcome. The second limitation was the exclusion of two studies assessing melatonin from the quantitative analysis owing to inadequate reporting of the change in depression scores. While more extensively evaluating the antidepressant effects of mitochondrial modulators in BD has been completed, lacking other studies assessing the effects of melatonin in the treatment of BD. In addition, the presence of potential bias in several of the included studies presents another limitation of the current analysis. Finally, all included trials had a relatively short duration. Long-term studies are further needed to examine whether mitochondrial modulators can prevent the recurrence of depressive symptoms in patients with BD.
